# Progenitor “*Mycobacterium canettii*” Clone Responsible for Lymph Node Tuberculosis Epidemic, Djibouti

**DOI:** 10.3201/eid2001.130652

**Published:** 2014-01

**Authors:** Yann Blouin, Géraldine Cazajous, Céline Dehan, Charles Soler, Rithy Vong, Mohamed Osman Hassan, Yolande Hauck, Christian Boulais, Dina Andriamanantena, Christophe Martinaud, Émilie Martin, Christine Pourcel, Gilles Vergnaud

**Affiliations:** Université Paris-Sud, Orsay, France (Y. Blouin, Y. Hauck, C. Pourcel, G. Vergnaud);; Centre National de la Recherche Scientifique, Orsay (Y. Blouin, Y. Hauck, C. Pourcel, G. Vergnaud);; Hôpital Militaire Bouffard, Djibouti, Republic of Djibouti (G. Cazajous, C. Dehan, C. Boulais);; Hôpital d'Instruction des Armées Percy, Clamart, France (C. Soler, R. Vong, C. Martinaud);; Hôpital Paul Faure, Djibouti (M. Osman Hassan);; Hôpital d'Instruction des Armées Bégin, Saint-Mandé, France (D. Andriamanantena);; Centre Hospitalier Lyon Sud, Lyon, France (E. Martin);; Institut de Recherche Biomédicale des Armées, Brétigny, France (G. Vergnaud)

**Keywords:** Mycobacterium, tuberculosis, lymph node tuberculosis, Mycobacterium canettii, disease outbreaks, Djibouti, tuberculosis and other mycobacteria

## Abstract

“*Mycobacterium canettii*,” an opportunistic human pathogen living in an unknown environmental reservoir, is the progenitor species from which *Mycobacterium tuberculosis* emerged. Since its discovery in 1969, most of the ≈70 known *M. canettii* strains were isolated in the Republic of Djibouti, frequently from expatriate children and adults. We show here, by whole-genome sequencing, that most strains collected from February 2010 through March 2013, and associated with 2 outbreaks of lymph node tuberculosis in children, belong to a unique epidemic clone within *M. canettii*. Evolution of this clone, which has been recovered regularly since 1983, may mimic the birth of *M. tuberculosis*. Thus, recognizing this organism and identifying its reservoir are clinically important.

## Abstract 2

Two outbreaks among expatriate children were caused by an epidemic clone from the Horn of Africa.

Most “*Mycobacterium canettii*” strains have been isolated in the Republic of Djibouti, where 2 hospitals manage tuberculosis (TB) infections among the Djiboutian population and expatriates (*1**,**2*). A study of clinical and epidemiologic data linked to *M. canettii* infections showed that the proportion of TB cases caused by *M. canettii* was higher among expatriate than among Djiboutian patients and that patients with *M. canettii* infection were significantly younger than those with *M. tuberculosis* infection (*2*). These findings suggested that the Djiboutian population had been immunized against infection by *M. canettii*. No difference was observed in the frequency of the nonpulmonary form of TB caused by *M. tuberculosis* or *M. canettii*.

*M. canettii* is the progenitor species from which *M. tuberculosis* emerged (*3**–**5*). Genotyping of known *M. canettii* isolates showed that 70% of them belong to a large cluster called A (*1**,**3*). Strains belonging to cluster A were isolated as early as 1983. This observation and the absence of human-to-human transmission support the existence of an environmental reservoir. We report the isolation, since 2010, of 21 new strains of *M. canettii* in Djibouti, of which 7 were associated with 2 lymph node TB outbreaks in children. We show that 17 of the new strains, including the outbreak strains, belong to cluster A. We use draft whole-genome sequencing to demonstrate that this cluster is remarkable among *M. canettii* strains and confirm its epidemic status, which suggests an accelerating emergence of a clone, subsequently called clone A. Within clone A, we identify a single horizontal genetic transfer event, presumably resulting from recombination with closely related mycobacteria. We also investigate CRISPRs (clustered regularly interspaced short palindromic repeats) because these structures, which keep a memory of past infections by bacterial viruses, may provide indirect clues about an environmental reservoir. We take advantage of the clone A sequence data, which is, within *M. canettii*, closest to *M. tuberculosis*, to better describe the emergence of *M. tuberculosis*.

## Materials and Methods

### Isolation and Culture

Most samples (sputum, biopsy, or puncture from lymph node; gastric fluid; esophagus; pericardium) came from patients hospitalized from February 2010 to March 2013 in the French Military Hospital Bouffard in Djibouti, Republic of Djibouti (Table 1). One additional sample came from a patient who had been living in Djibouti for 2 years and was hospitalized in the University Hospital in Lyon, France, in August 2011. The samples were collected during the usual care of these patients, and the study was approved by the hospitals’ ethics committees.

**Table 1:** Characteristics of patients from whom *Mycobacterium canettii* isolates were obtained, Djibouti, 2010–2013*

Strain no.	Patient nationality (length of stay, mo)	Hospital	Sex	Isolation date	Age, y†	Sample	TB site	HIV status (CD4/mm^3^)	Cluster
Percy975	Djiboutian	Bouffard	M	2010 Feb	28	GF	Pulmonary	Pos (122)	A‡
Percy976	Djiboutian	Bouffard	M	2010 Feb	18	GF	Pulmonary	Neg	A
Percy977	Diboutian	Bouffard	F	2010 Feb	22	GF	Pulmonary	Neg	A
Percy979	Djiboutian	Bouffard	F	2010 Feb	39	GF	Pulmonary	Neg	A
Percy1004	Djiboutian	Bouffard	M	2010 Jun	14	LN puncture	LN	Neg	Singleton‡
Percy1049	Ethiopian (18)	Bouffard	F	2011 Jan	36	GF	Pulmonary	Pos (9)	A
Percy1060§	Djiboutian	Bouffard	M	2011 Feb	33	Sputum	Diffuse	Pos (235)	A
Percy1062	French (8)	Bouffard	M	2011 Mar	40	GF	Pulmonary	Neg	C
Percy1064	Djiboutian	Bouffard	M	2011 Mar	55	Sputum	Pulmonary	Neg	C
Percy1077	French (12)	Bégin	M	2011 Jul	48	Esophagus biopsy	Esophagus	Pos (UNK)	A
Percy1078	French (13)	Bouffard	F	2011 Sep	3	LN puncture	LN	Neg	A‡
Percy1079	French (13)	Bouffard	M	2011 Sep	1	LN biopsy	LN	Neg	A
Percy1084	French(3)	Bouffard	M	2011 Oct	4	LN puncture	LN	Neg	A‡
Percy1085	French (24)	Lyon	F	2011 Aug	8	LN biopsy	LN	UNK	A
Percy1086	Djiboutian	Bouffard	M	2012 Jan	51	Pericardium biopsy	Diffuse	Pos (52)	A
Percy1101	Djiboutian	Bouffard	F	2011 May	26	GF	Pulmonary	Neg	C‡
Percy1105	French (15)	Bouffard	M	2012 Oct	44	GF	Pulmonary	Neg	A‡
Percy1115	French (4)	Bouffard	M	2012 Dec	3	LN biopsy	LN	Neg	A‡
Percy1116	French (5)	Bouffard	M	2012 Dec	12	LN puncture	LN	Neg	A‡
Percy1129	French (42)	Bouffard	F	2013 Jan	11	LN puncture	LN	Neg	A‡
Percy1130	Djiboutian	Bouffard	M	2013 Mar	35	GF	Pulmonary	Pos (122)	A‡

*TB, tuberculosis; GF, gastric fluid; LN, lymph node; Diffuse, pulmonary and extrapulmonary; Pos, positive; Neg, negative; UNK, unknown. †Age at isolation date. ‡Ten of the 17 strains selected for draft genome sequencing. §A second isolate, Percy1050, recovered from a lymph node biopsy specimen, showed the same multiple-locus variable number tandem repeat analysis genotype.

Of the 22 samples (including 2 samples from the same patient), 10 were processed on site, 1 in Lyon, and the last 11 at the Percy Military Hospital (Clamart, France). After samples were decontaminated by sodium hydroxide (NaOH) in N-acetyl-L-cysteine-sodium hydroxide (NALC-NaOH method) (*6*), cultures were done on solid medium (Lowenstein-Jensen) and also in liquid medium for samples sent to France. Susceptibility of the isolates to drugs was measured in liquid medium (BACTEC 960, Becton Dickinson, Le Pont de Claix, France). Identification of the species was made by rapid chromatographic lateral flow assays (SD Bioline TB Ag MPT64 Rapid, Standard Diagnostics, Gyeonggi-Do, South Korea), the DNA strip assay GenoType MTBC (Hain Lifescience, Nehren, Germany), and biochemical analyzes. *M. canettii* strains Percy22, Percy50, and Percy975 were previously described (*1*). Strains were genotyped by using 24 tandem repeat loci (*1*).

### Draft Whole-Genome Sequencing and in silico Analysis

The genome of selected strains was sequenced on the HiSeq2000 or MiSeq Illumina platform (BaseClear, Leiden, the Netherlands, or Imagif, Gif-sur-Yvette, France). Raw sequence data files were deposited in the European Nucleotide Archive (ENA project accession no. ERP002514), maintained by the European Bioinformatics Institute.

Single-nucleotide polymorphisms (SNPs) were determined by alignment with reference strains (*M. tuberculosis* H37Rv accession no. NC_000962.3 or *M. canettii* cluster A Percy3 [STB-D CIPT140060008 accession no. NC_019950.1]) as described (*7*; Technical Appendix 1). The determination of statistically significant clustering of polymorphic positions was done essentially as described by Croucher et al. (*8*). 

A de novo assembly was performed to produce draft genomes. The resulting contigs and additional published *M. canettii* sequence data were compared with *M. tuberculosis* genomes to identify regions that would be shared by all sequenced *M. canettii* strains but absent from *M. tuberculosis* strains.

Four different types of CRISPR loci were previously identified in *M. canettii* (*5*) and called III-A, I-C, I-Cvar, and I-E (Table 2; Technical Appendix 2 Table 1). The *M. tuberculosis* CRISPR locus belongs to type III-A. To search for additional CRISPR loci potentially present in the new strains, CRISPRfinder analysis was applied to the draft genome assemblies (*10*). The CRISPRtionary tool was used to compare CRISPR sequence data (*11*).

**Table 2:** Sequence analysis of CRISPRs alleles of the *Mycobacterium canettii* isolates, Djibouti, 2010–2013*

Cluster	Strain	No. spacers	Allele code†	Alias‡ (accession no.)
A	Percy3 and all other clone A strains	26	III-A-69@94	STB-D CIPT140060008 (NC_019950)
C	Percy1004	12	III-A-69@74-95-96-89@91-94	
C	Percy32	30	III-A-69@74-95@98-75@91-94	
C	CIPT140010059	29	III-A-69@74-95@98-75@91-94	STB-A CIPT140010059 (NC_015848)
–	Percy79	31	III-A-99@129	
–	Percy301	31	III-A-99@129	
B	Percy214	8	I-C-130@137	STB-H CIPT140070013
B	Percy525	8	I-C-130@137	
C	Percy1101	14	I-C-130@143	
–	Percy25	7	I-C-131-132-144@148	STB-E CIPT140070002
–	Percy65	27 (1 doublet)	I-C-201@205-203-206@226	STB-J CIPT140070017 (NC_019952)
–	Percy327	9	I-C-130-133@137-198@200	STB-L CIPT140070008 (NC_019965)
–	Percy302	50; 53	I-C-149@191-178var-192@197;I-Cvar-337@389	STB-K CIPT140070010 (NC_019951)
–	Percy89	83	I-E-228-230@232-88var-233@240-245@258-278@333	STB-G CIPT140070005
–	Percy99b	58	I-E-227@232-88var-233 @280-334@336	STB-I CIPT140070007
–	Percy157	52	I-E-227@232-88var-233@277	

*CRISPR, clustered regularly interspaced short palindromic repeats. †The CRISPR type is indicated as prefix (type III-A, I-C, I-Cvar, I-E). Allele codes refer to the spacers dictionary (Technical Appendix 2 Table 1). "69@94" indicates that all spacers from 69 to 94 are present. Spacers 1 to 68 have been previously reported in *M. tuberculosis *(*9*). Spacer numbering in "*M. canettii*" runs from 69 up to 389, i.e., the total number of spacers observed in *M. canettii* is 321. ‡Strains sequenced by (*5*) as draft or completed (European Nucleotide Archive accession no. indicated) genome 88var = TCCAGAGGTCGAAGTGATGTTCGGTGTTCTCCT.

## Results

### Epidemiologic Investigation

During February 2010–March 2013, a total of 240 cases of TB were diagnosed in Bouffard Military Hospital (220 Djiboutian and 20 non-Djiboutian patients, including 13 children [patients <15 years]). *M. canettii* was isolated from 21 patients, representing 8.7% of all cases: *M. canettii* was responsible for 4.4% of TB cases in Djiboutians (10 patients) and 55% of TB cases in non-Djiboutians (11 patients). Ten patients had pulmonary TB, 9 had extrapulmonary TB, and 2 showed disseminated infections. The patients were predominantly male (14 male, 7 female) and young (mean age 25.2 years; range 1–55 years). The clinical, biological, and radiologic data did not differ from data from the other patients who had TB diagnosed in this hospital (Table 1). All children had lymph node TB, and conversely, all lymph node TB cases were observed in children.

Nine of the 10 Djiboutian patients with *M. canettii* infection were adults, including 7 case-patients with pulmonary TB (2 persons were HIV positive) and 2 case-patients with disseminated TB (both HIV positive). The last Djiboutian patient was 14 years old and had lymph node TB. The mean age was 31.3 years (range 14–55 years).

The 11 other patients were expatriates (10 from France, 1 from Ethiopia; 2 were HIV positive) with an average duration of stay in Djibouti of 13.8 months (range 3–42 months) (Table 1). Four were adults (3 with pulmonary TB, 1 with extrapulmonary TB [esophagus]; 2 were positive for HIV; age range 36–48 years), and 7 were children (age range 1–12 years). Four cases occurred from August to October 2011 and the last 3 occurred during December 2012–January 2013 (with another 2 suspected cases from which no bacteria could be isolated). All patients had received bacillus Calmette–Guérin vaccine. Inquiries were made concerning each case-patient (within the family, home workers, or at school/work), but no contagious or infected person could be identified. From the beginning of February 2010 through the end of March 2013, a total of 1,661 French children came to Djibouti, according to the French consulate. This provides an estimated probability of declaring a *M. canettii* infection of ≈0.5%.

All isolates were tested and found to be sensitive to rifampin, isoniazid, pyrazinamide, and ethambutol. The expatriate patients were treated at the Bouffard Military Hospital, Bégin Military Hospital, or Lyon Hospital. The 4 adults were successfully treated by rifampin/isoniazid/pyrazinamide/ethambutol for 2 months and then received rifampin/isoniazid for 4 months. The 7 children received the same classic treatment procedure without ethambutol, with 2 exceptions in which ethambutol was added in the second month of treatment because of the enlargement of the first lymph node and appearance of a second lymph node. One of these 2 patients, a 3-year-old child, had surgery 10 months later. For the other patient, the treatment was successful after 6 months.

### Genotyping New *M. canettii* Strains and Selecting Strains for Draft Sequencing

The genotypes of the 22 new isolates were compared to published data, which showed that 18 belong to the previously described cluster A (including the 2 isolates derived from the same patient; data not shown) (*1*). Percy1062, Percy 1064 and Percy1101, together with Percy32 and 2 historical *M. canettii* strains (CIPT140010060, CIPT140010059), belong to the much smaller and more diverse cluster C (*1*). Percy1004 is more distant.

A total of 17 *M. canettii* strains were selected for draft whole-genome sequencing including 10 cluster A strains (8 strains recovered since 2010 [Table 1] and strains Percy50 and Percy22, collected in 1983 and 2003, respectively) and 7 genetically diverse strains (Percy32, Percy79, Percy157, Percy301, Percy525, Percy1004, Percy1101). Percy302, which was previously fully sequenced under the name STB-K and was shown to be the most remote *M. canettii* strain (*5*), was included for draft re-sequencing as a control. The sequences of these strains were analyzed, together with those of 10 strains previously described (*5**,**11*), representing a total of 27 *M. canettii* strains.

### Whole-Genome SNP Analysis

During analysis of all sequenced *M. canettii* and *M. tuberculosis* genomes, 75,412 SNPs were determined, compared with the 13,358 identified within the *M. tuberculosis* complex alone (*7*) (Technical Appendix 1). The 2 independent sequence datasets for Percy302 (STB-K) clustered closely together (7 differences) as expected. The mean divergence between *M. canettii* isolates was an average of 10 times that inside *M. tuberculosis*, in agreement with previous reports (*4**,**5*). The clustering achieved by single nucleotide polymorphism analysis was in good agreement with the genotyping data. For instance, cluster B and cluster C strains were similarly grouped by both approaches. The clustering of A strains was most remarkable. K116, which was independently investigated (*12*) and for which no genotyping data were available, also belongs to cluster A. This homogeneity is remarkable because cluster A included strains isolated during 1983–2013 (Technical Appendix 1).

### SNP Analysis within Cluster A

**Figure 1:**
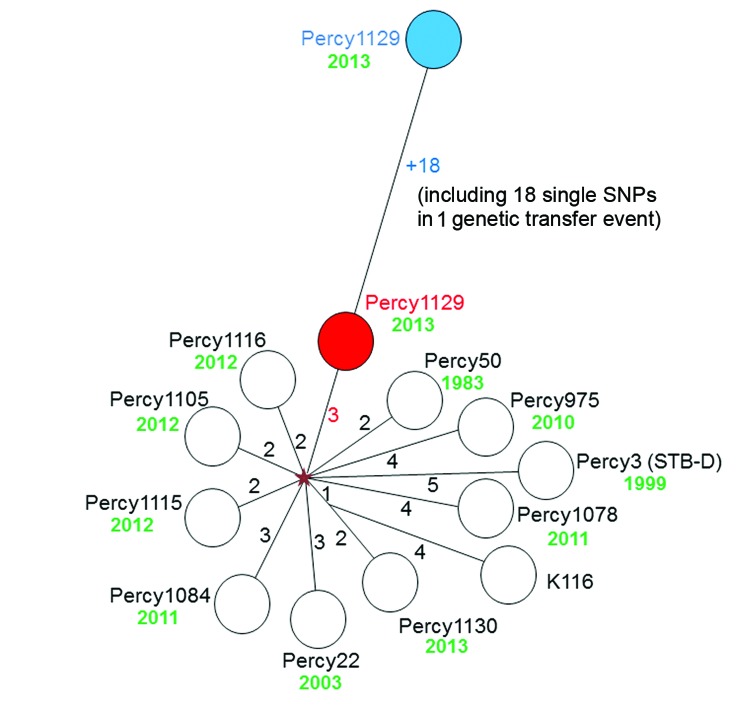
Starburst genealogy within clone A of *Mycobacterium canettii* isolates, Djibouti, 2010–2013. The size of each branch, corresponding to the number of polymorphisms between 2 nodes, is indicated. The tree is based upon 55 polymorphisms, 18 of which are clustered in 1,660 base pairs. The relative position of Percy1129 is shown with (blue) or without (red) these 18 polymorphisms. The isolation year is indicated near each strain. The position of a hypothetical ancestor is indicated by the red. All cluster A strains are 2 up to a maximum of 5 polymorphisms away from this hypothetical ancestor after removal of the exceptional polymorphism cluster found in strain Percy1129. SNP, single-nucleotide polymorphism.

A total of 55 SNPs were identified among the 12 cluster A strains by alignment on the fully sequenced genome of cluster A strain Percy3 (STB-D; NC_019950; Table 2). A minimum-spanning tree was drawn (Figure 1). There was no homoplasy in this tree, indicating that these single nucleotide polymorphisms did not appear twice independently within this group of strains. The distribution of the polymorphisms along the reference genome was analyzed to detect abnormal densities, potentially resulting from horizontal gene transfer by homologous recombination. Notably, a single instance could be identified. Eighteen polymorphisms fell within a single cluster covering 1,660 bp observed in Percy1129, compared with the other cluster A genomes. This 1% sequence divergent segment covers 2 full genes (Technical Appendix 2, Table 2). The ratio of nonsynonymous to synonymous SNPs is strikingly different in the 2 groups, consistent with previous observations (*13**–**15*). The ratio is low among the group of clustered SNPs, and remarkably high among the group of unclustered polymorphisms (Technical Appendix 2 Table 2). Figure 1 shows (blue) the initial position of Percy1129 and its position after removal of this unique genetic transfer event (red). There was no obvious correlation between branch length and strain isolation date. In contrast to the B and C clusters, the A cluster strains clearly belong to an epidemic clone and will subsequently be called clone A.

### Rooting the *M. tuberculosis* Phylogenetic Tree

**Figure 2:**
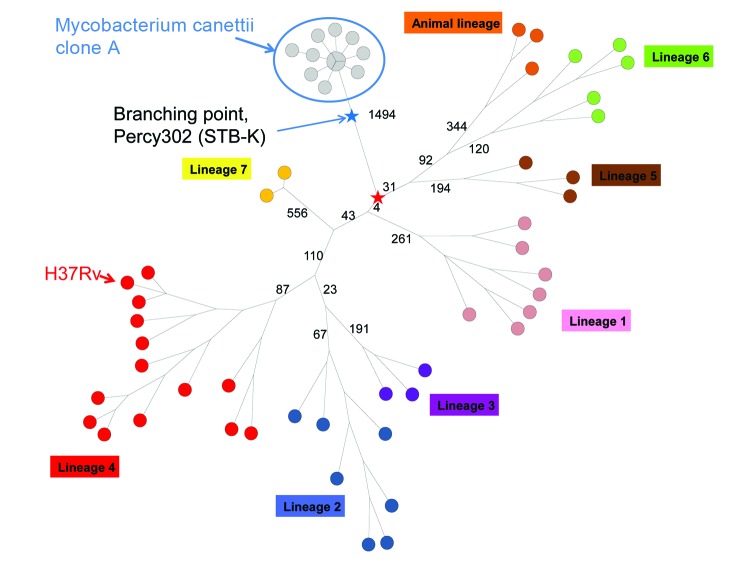
Early evolution of *Mycobacterium tuberculosis* was deciphered using clone A sequence data. A minimum spanning tree was drawn after removal of polymorphisms occurring in clusters, indicative of horizontal gene transfer events. The approximate position of the branching point of Percy302 (STB-K) the most distantly related *M. canettii* strain (*5*) is indicated by the blue star. The red star is the position of the most recent common ancestor of *M. tuberculosis*. The branch lengths of only the most internal branches are indicated. Branch length values inside clone A are <3. The position of the reference strain H37Rv is indicated. Four hundred seventy-one polymorphisms separate the red star from H37Rv. A logarithmic branch length representation was used.

Among *M. canettii* strains, clone A was previously shown to be the closest to *M. tuberculosis* in terms of shared ancestry (*5*). Consequently, clone A sequence data constitute the current best resource to root *M. tuberculosis* (*7*). We merged the list of SNPs reported within *M. tuberculosis* (*7*) with additional polymorphisms deduced from the alignment of the clone A strains on H37Rv to produce a minimum-spanning tree showing precisely the *M. canettii* branching point (red star in Figure 2). The branch containing 4 polymorphisms in Figure 2 demonstrates that the *M. tuberculosis* superlineage containing *M. africanum* and *M. bovis* was the first extant lineage to emerge from the cradle of *M. tuberculosis* in the Horn of Africa (*7*). The blue star indicates the position of the node leading to Percy302 (STB-K), the most genetically diverse *M. canettii* strain. This branching point is significantly closer to clone A than to the red star, indicating a faster mutation rate along the branch leading to the red star, potentially more similar to that observed within *M. tuberculosis*. This might provide indirect evidence for a substantial ecologic change well before this branching point, i.e., a speciation event of *M. tuberculosis* preceding the most recent common ancestor defined by extant lineages.

### In silico Study of CRISPR Locus

A single CRISPR type was found in each genome as previously observed, except for Percy302 (STB-K), which contains 2 CRISPR structures, I-C and I-Cvar, comprising 50 and 53 spacers, respectively (*5*). The largest CRISPR allele was found in Percy89 (STB-G), with 83 spacers in its type I-E CRISPR. All clone A strains, including K116, possess an identical type III-A locus composition. Strain Percy1101 belongs to cluster C (Technical Appendix 1), but its CRISPR structure, type I-C, was different from that of strains of this group (associated with III-A). A total of 321 spacers were detected in the present “*M. canettii*” collection (Table 2; Technical Appendix 2 Table 1). Locus III-A contributed 61 spacers, locus I-C 97 spacers, locus I-C var 53 spacers, and locus I-E 110 spacers. Three independent events of spacer acquisition from the same source were identified, resulting in only slightly different spacer composition in different CRISPR alleles (Table 2; Technical Appendix 2 Table 1). Nine spacers matched a prophage in *M. marinum* strain M (within positions 4,821,000 and 4,847,000 of accession no. CP000854.1). Two others matched *Mycobacterium* phages Thibault or Redi. One spacer in the Percy25 (STB-E) type I-C CRISPR allele matched perfectly 36 bp in gene *aftB* (locus tag Rv3805c in H37Rv) (Technical Appendix 2 Table 1).

### Absence of Part of Vitamin B12 Synthesis Pathway in *M. tuberculosis*

One particular region of interest was shown to be specific of the *M. canettii* taxon compared with that of *M. tuberculosis*. This region, which encompassed 3 kb on the Percy3 (STB-D) genome, from position 1,048,604 to position 1,050,991, contains the *cobF* (precorrin) gene and is present in all *M. canettii* strains, although it is absent from all *M. tuberculosis* genomes. This gene is part of a vitamin B12 synthesis pathway, suggesting that this pathway is nonfunctional in *M. tuberculosis*.

## Discussion

The prevalence of *M. canettii* in TB patients in the Republic of Djibouti is unique, with >8% of cases reported to Bouffard Hospital during 2010 through early 2013 caused by *M. canettii*. In our experience, *M. canettii* is more frequently the cause of TB among expatriates (particularly children) and severely immunodepressed HIV-positive patients. However, the proportion of *M. canettii* infections is probably biased because the patients consulting at Bouffard Hospital are very likely not representative of the general population. For instance, all French TB patients were treated in Bouffard, and about half were infected by *M. canettii*. Not including the expatriates, the prevalence of *M. canettii* infection is 4%, which is still remarkably high. This raises the possibility that the prevalence of *M. canettii* is underestimated in the population of TB patients in Djibouti, or that additional bias exists in terms of socioeconomic background in the population of TB patients seeking treatment at Bouffard Hospital (*16*). Notably, all infected children had lymph node TB, and all cases of lymph node TB were observed in children. This calls for better surveillance of enlarged lymph nodes in children. *M. canettii* reservoirs likely are not strictly restricted to Djibouti but can be found in neighboring countries and in other large multicultural cities.

Clone A strains constitute an emerging pathogenic clone that appears to be much more successful at infecting humans than are other *M. canettii* representatives, because an almost identical strain has been predominantly isolated over the last 3 decades and represents 70%–80% of all *M. canettii* strains. The 2 outbreaks of lymph node TB reported in 2011 and 2012–2013, mainly in young children, raise again the question of the reservoir for this pathogen and this particular clone, and the reason for its increased virulence. In a mouse model, a clone A strain persisted longer in the lungs than any of the other *M. canettii* strains tested (*5*). This result could explain the difficulties in treating 2 of the young patients. However, for these 2 patients, the extension of lesions might also be the result of a paradoxical upgrading reaction or resistance of the strain to antimicrobial drugs used. Indeed, it was previously reported that *M. canettii* was more resistant in vitro to pyrazinamide and pyrazinoic acid than was *M. tuberculosis* (*2**,**17**,**18*). These points will deserve further investigations. The frequency at which clone A strains infect humans may also reflect a higher success in colonizing a reservoir with which persons in Djibouti are in closer contact. Some of the expatriate patients had been living in Djibouti for short periods of time (4 months for the youngest 3-year-old patient). Although attempts to isolate *M. canettii* from the environment and animals in contact with the infected children have not been successful thus far, efforts in this direction should clearly be reinforced.

Fifty-five SNPs were identified by comparison among the sequenced clone A strains, 18 of which could be linked to a single horizontal gene transfer event with an unknown closely related mycobacterium. It was recently shown in *M. smegmatis* that distributive conjugal transfer could induce multiple genetic transfer events in a single step, and the authors of that study proposed that this mechanism created the genome mosaicism observed among *M. canettii* (*19*). Our observation of a unique transfer event does not support this hypothesis or would suggest that, in *M. canettii*, conjugal transfer is not associated with multiple events.

When only new mutational events are taken into account, the proportion of nonsynonymous mutations and, most notably, the branch lengths within clone A are typical of an *M. tuberculosis* outbreak (*7**,**20**,**21*). The expansion of clone A is thus likely to be very recent. The horizontal gene transfer events can only be explained by the existence of *M. canettii* in a reservoir or inside hosts such as the amoeba (*22*) in which *M. canettii* strains can exchange DNA with other *M. canettii* strains or with closely related mycobacteria that are not infectious for humans.

### Links between *M. canettii* Clone A and the *M. tuberculosis* Complex

The finding that only 4 SNPs separate the radiation of *M. tuberculosis* lineages 5–6 and that of lineage 1 suggests that their diversification could correspond to a unique outbreak event, because this distance is consistent with observations of the accumulations of such polymorphisms during an outbreak (*7**,**20*). Along this line, it is tempting to speculate that clone A is reproducing the early steps which led to the speciation of *M. tuberculosis*. This may be favored by a situation in which a relatively naive population, in terms of exposure to *M. tuberculosis* (children and expatriates), is being exposed to the environmental reservoir. Eventually a strain with the appropriate mutation might spread from human to human. 

We have been able to identify in clone A 1 horizontal gene transfer event with non–clone A strains or more likely a non–*M. canettii* mycobacterium, presumably occurring in the environment. If the ability of *M. tuberculosis* to spread had been acquired in the environment rather than in the human host, then there would be a possibility that different *M. tuberculosis* lineages emerged independently from its reservoir. These different lineages might be distinguished by traces of ancestral horizontal gene transfer events, visible in the very internal branches of *M. tuberculosis* evolution, as observed here within clone A. We could not identify any such fossils of early horizontal gene transfer events, which is compatible with a model in which the most recent ancestor of *M. tuberculosis* never lived in the environment. One possibility is that it acquired a key feature leading to speciation during the colonization of its human host, after infection from the environment. Another possibility is that the most recent ancestor of *M. tuberculosis* does not coincide with the speciation of *M. tuberculosis* the obligatory human pathogen (*23*) as suggested here by comparing evolutionary rates toward *M. canettii* clone A and toward *M. tuberculosis*. Clone A may mimic an earlier phase before *M. tuberculosis* speciation. Speciation, associated with the ability to spread from human to human and not only the capacity to cause TB, which is clearly much more ancient, would have resulted from the multiple events of human TB infections caused by *M. canettii*, interspersed with genetic reshuffling of *M. canettii* in the environment. We hope that the list of polymorphisms identified in this investigation will facilitate the analysis of ancient *M. tuberculosis* and allow better positioning of *M. tuberculosis* speciation with respect to its current most recent common ancestor.

### CRISPR Diversity

Within the investigated *M. canettii* strains, >300 spacers can be identified. Only a few show significant similarity with sequences in the GenBank nonredundant nucleotides section. One spacer found in a single *M. canettii* strain matches a chromosomal gene, as often seen in *Yersinia pestis* CRISPRs (*24*), suggesting that this chromosomal locus may be the subject of CRISPR interference in this particular strain. Notably, the other matches are with *Mycobacterium* phages, including a *M. marinum* prophage, which may indicate an aquatic reservoir for *M. canettii*.

Technical Appendix 1 Supplement describing the identification and validation of single nucleotide polymorphisms, their de novo assembly, and the identification and elimination of regions with higher density; a neighbor-joining dendrogram of polymorphisms shows clustering for 72 strains.

Technical Appendix 2 Two tables showing 1) a full list of spacers identified in *Mycobacterium canettii* and *M. tuberculosis* and 2) a list of 55 single nucleotide polymorphisms identified within clone A.
