# Supplementary material for: Progenitor “Mycobacterium canettii” Clone Responsible for Lymph Node Tuberculosis Epidemic, Djibouti
Source: Emerg Infect Dis. 2014 Jan;20(1):21–8. doi: 10.3201/eid2001.130652 (PMC3884719; doi:10.3201/eid2001.130652)
Supplement: Technical Appendix 1 — Supplement describing the identification and validation of single nucleotide polymorphisms, their de novo assembly, and the identification and elimination of regions with higher density; a neighbor-joining dendrogram of polymorphisms shows clustering for 72 strains. [file 13-0652-Techapp-s1.pdf]

Article DOI: http://dx.doi.org/10.3201/eid2001.130652

Progenitor "*Mycobacterium canettii*" Clone Responsible for Lymph Node Tuberculosis Epidemic, Djibouti

Technical Appendix 1

Single Nucleotide Polymorphisms (SNPs) Identification and Validation

SNPs were identified essentially as previously described (*1*–*3*). For each strain, sequence reads were mapped on the reference genome to produce a homology assembly using BioNumerics version 7.1 (Applied-Maths, Belgium). The parameters for mapping were a minimum sequence identity of 75% for the alignment of the reads with the reference genome, and a minimum coverage of 10 for each base. The reconstructed genomes were adjusted in order to start at the same position and thus be perfectly colinear to the reference genome. They were then scanned to identify SNPs with a known status in all genomes using a homemade Python script. SNPs which belonged to homologous gene families (such as the PE and PPE gene families) were removed.

De novo Assembly

De novo assembly was performed using Velvet (*4*) as embedded within the Power Assembler module of BioNumerics version 7.1. The length of the k-mer was set to 31, which provided the best results, both in terms of contigs numbers (fewer contigs) and in terms of the total number of bases produced (longer contigs).

Identification and Elimination of Regions with Higher SNP Density

SNPs were analysed to produce a minimum spanning tree allowing the creation of hypothetical missing links using BioNumerics version 7.1. The position on the reference genome of the SNPs present within each branch was analysed as described by (*5*) in order to identify statistically significant high concentrations of SNPs. The corresponding SNPs were

filtered out from the SNP dataset, and the process was repeated until no more significant SNP clusters were identified.

Technical Appendix 1 Table. Main features of the raw sequence data

Strain	Average mapped sequencing depth	Reads size	No. reads mapped on STB-D	Unambiguous bases	Genome coverage (%)
K116	136.53	75	7953491	4368961	98.57
Percy50	146.62	75	8598811	4398401	99.23
Percy975	169.97	75	9966578	4397707	99.22
Percy22	328.63	100	14523151	4419346	99.7
Percy1078	145.99	100	6436928	4409042	99.47
Percy1084	96.54	100	4248792	4400955	99.29
Percy1105	524.87	250	9284619	4422372	99.77
Percy1115	134.24	250	2356950	4389349	99.03
Percy1116	93.06	250	1625649	4366980	98.52
Percy1129	33.22	250	583950	4394967	99.15
Percy1130	24.47	250	420363	4294759	96.89

Technical Appendix 1 Figure (following page). Neighbor-joining dendrogram of polymorphisms clustering for 72 strains, including 27 *Mycobacterium canettii* and 45 strains belonging to the *M. tuberculosis* complex, with no filtering of clustered single nucleotide polymorphisms. Percy65 (STB-J) and Percy302 (STB-K) were used to root the tree (*6*). A star after a strain name indicates strains sequenced for this study. Clone A and clusters B and C are boxed. Lineages within *M. tuberculosis* are indicated using the same color code as in Figure 2. Cluster cophenetic correlation values <100 are indicated.

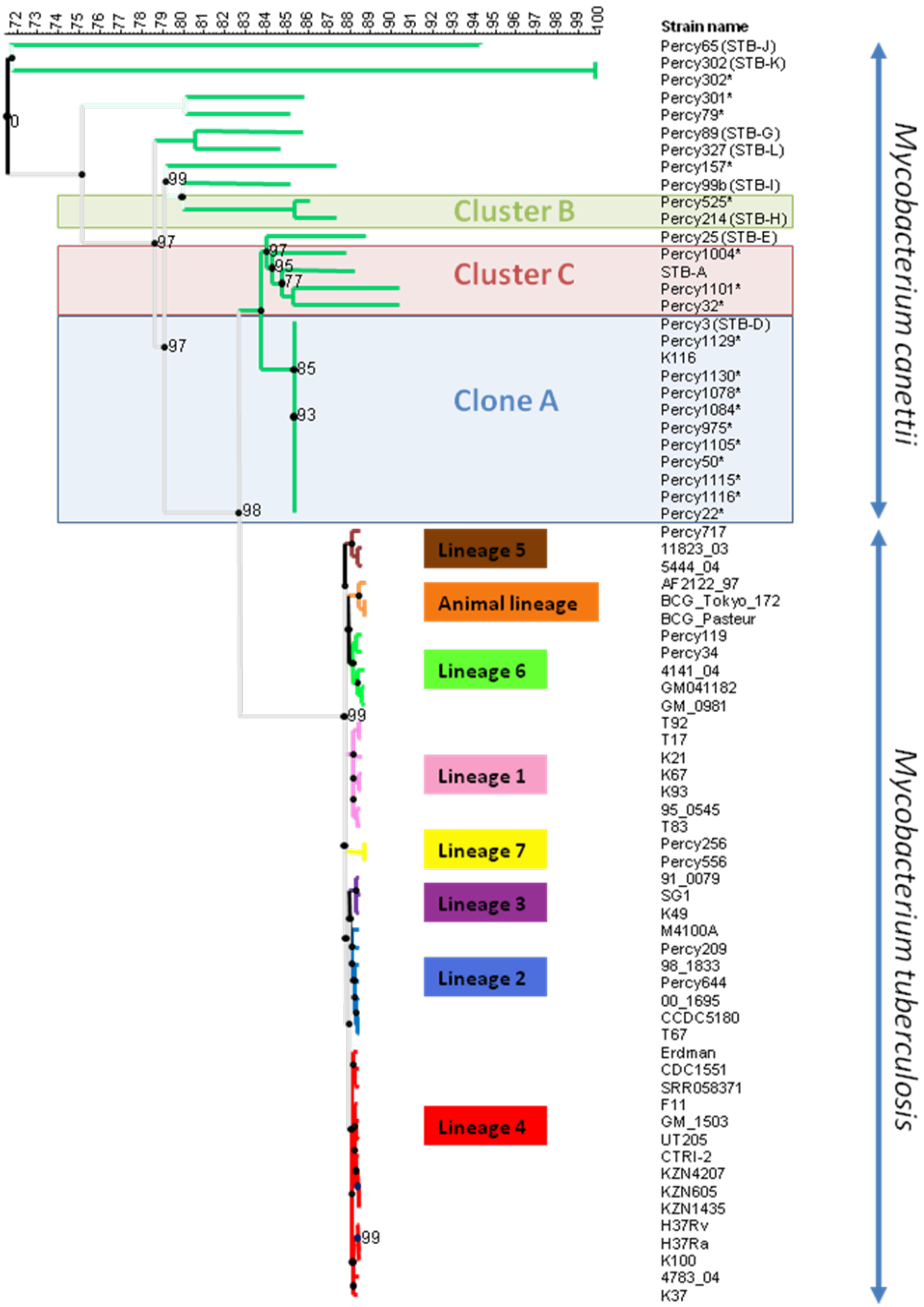
72 73 74 75 76 77 78 79 80 81 82 83 84 85 86 87 88 89 90 91 92 93 94 95 96 97 98 99 100
Strain name
Percy65 (STB-J)
Percy302 (STB-K)
Percy302*
Percy301*
Percy79*
Percy89 (STB-G)
Percy327 (STB-L)
Percy157*
Percy99b (STB-I)
Percy525*
Percy214 (STB-H)
Percy25 (STB-E)
Percy1004*
STB-A
Percy1101*
Percy32*
Percy3 (STB-D)
Percy1129*
K116
Percy1130*
Percy1078*
Percy1084*
Percy975*
Percy1105*
Percy50*
Percy1115*
Percy1116*
Percy22*
Percy717
11823_03
5444_04
AF2122_97
BCG_Tokyo_172
BCG_Pasteur
Percy119
Percy34
4141_04
GM041182
GM_0981
T92
T17
K21
K67
K93
95_0545
T83
Percy256
Percy556
91_0079
SG1
K49
M4100A
Percy209
98_1833
Percy644
00_1695
CCDC5180
T67
Erdman
CDC1551
SRR058371
F11
GM_1503
UT205
CTRI-2
KZN4207
KZN605
KZN1435
H37Rv
H37Ra
K100
4783_04
K37
Cluster B
Cluster C
Clone A
Lineage 5
Animal lineage
Lineage 6
Lineage 1
Lineage 7
Lineage 3
Lineage 2
Lineage 4
Mycobacterium canettii
Mycobacterium tuberculosis

References

1. Blouin Y, Hauck Y, Soler C, Fabre M, Vong R, Dehan C, et al. Significance of the identification in the Horn of Africa of an exceptionally deep branching *Mycobacterium tuberculosis* clade. PLoS ONE. 2012;7:e52841. PubMed http://dx.doi.org/10.1371/journal.pone.0052841

2. Kuroda M, Serizawa M, Okutani A, Sekizuka T, Banno S, Inoue S. Genome-wide single nucleotide polymorphism typing method for identification of *Bacillus anthracis* species and strains among *B. cereus* group species. J Clin Microbiol. 2010;48:2821–9. PubMed http://dx.doi.org/10.1128/JCM.00137-10

3. Ford C, Yusim K, Ioerger T, Feng S, Chase M, Greene M, et al. *Mycobacterium tuberculosis* - Heterogeneity revealed through whole genome sequencing. Tuberculosis (Edinb). 2012;92:194–201. PubMed http://dx.doi.org/10.1016/j.tube.2011.11.003

4. Zerbino DR, Birney E. Velvet: algorithms for *de novo* short read assembly using de Bruijn graphs. Genome Res. 2008;18:821–9. PubMed http://dx.doi.org/10.1101/gr.074492.107

5. Croucher NJ, Harris SR, Fraser C, Quail MA, Burton J, van der Linden M, et al. Rapid pneumococcal evolution in response to clinical interventions. Science. 2011;331:430–4. PubMed http://dx.doi.org/10.1126/science.1198545

6. Supply P, Marceau M, Mangenot S, Roche D, Rouanet C, Khanna V, et al. Genomic analysis of smooth tubercle bacilli provides insights into ancestry and pathoadaptation of *Mycobacterium tuberculosis.* Nat Genet. 2013;45:172–9. PubMed http://dx.doi.org/10.1038/ng.2517
